# Myoid differentiation in dermatofibrosarcoma protuberans and its fibrosarcomatous variant: 10 years’ experience in a tertiary hospital

**DOI:** 10.4322/acr.2021.368

**Published:** 2022-04-01

**Authors:** Chun-hai Lo, Po-man Tsang, Shui-ying Cheng

**Affiliations:** 1 CUHK Medical Centre, Department of Pathology, Hong Kong, China; 2 United Christian Hospital, Department of Pathology, Hong Kong, China

**Keywords:** Dermatofibrosarcoma, Antigens, CD34, Muscle, Smooth

## Abstract

Dermatofibrosarcoma protuberans (DFSP) is a relatively rare, locally aggressive, and dermal-based fibroblastic tumor. There are several histological variants, in which the usual emphasis is on fibrosarcomatous DFSP, as it acquires metastatic potential. Myoid differentiation in DFSP is rare, and more often found in fibrosarcomatous DFSP. Myoid differentiation is defined as tumor cells with brightly eosinophilic cytoplasm, well-defined cytoplasmic margins, and vesicular nuclei. In this study, we aim at characterizing the immunostaining pattern regarding myoid differentiation in DFSP, and discuss the potential pitfall in making the diagnosis. A total of ten cases of DFSP were found in the past ten years in our hospital. Two of them show focal myoid differentiation, including the only case of fibrosarcomatous DFSP. Around 5% of the tumor area in the traditional DFSP case shows myoid differentiation, while around 10% of the tumor area in fibrosarcomatous DFSP shows myoid differentiation. The myoid areas show positive staining, albeit patchy to focal, for smooth muscle markers, including smooth muscle actin, muscle-specific actin, caldesmon, and calponin. Staining for CD34, in those areas, is weak or negative. This may create diagnostic difficulty with smooth muscle tumors or myofibroblastic lesions, especially in a small biopsy sample. In difficult cases, the detection of COL1A1-PDGFB fusion by fluorescence in situ hybridization is helpful, as this is a characteristic chromosomal translocation found in the large majority of DFSP.

## INTRODUCTION

Dermatofibrosarcoma protuberans (DFSP) is a relatively rare, locally aggressive, and dermal-based fibroblastic tumor,^1^ with an incidence of two to four new cases per million per year.^2^^,^^3^ It usually affects young to middle-aged adults, with a slight male predominance. However, cases presenting in childhood and at birth have also been documented.^4^^-^7 It is most commonly found on the trunk and proximal extremities. Less commonly affected areas include the head and neck, genital area, breast, and acral sites.^8^^-^11 It typically presents as a slow-growing nodular or multinodular cutaneous mass with red to bluish discoloration. Early lesions may show a plaque-like growth with peripheral red discolouration. Rapid enlargement may occur during pregnancy or due to tumor progression to fibrosarcomatous DFSP.

There are several histological variants of DFSP, including pigmented DFSP (also known as Bednar tumor), myxoid DFSP, DFSP with myoid differentiation, plaque-like DFSP, and fibrosarcomatous DFSP. The usual emphasis is on fibrosarcomatous DFSP as it acquires metastatic potential. Myoid differentiation is rare and more often found in fibrosarcomatous DFSP.^12^^,^^13^

In this study, we characterize the immunostaining pattern regarding myoid differentiation in DFSP, and discuss the potential pitfall in making the diagnosis.

## STUDY

A total of seventeen cases of DFSP was found in the past ten years in the United Christian Hospital, Hong Kong, seven of which were excluded as those were biopsy or re-excisional specimens from the same tumor or patient.

**Figure 1:**
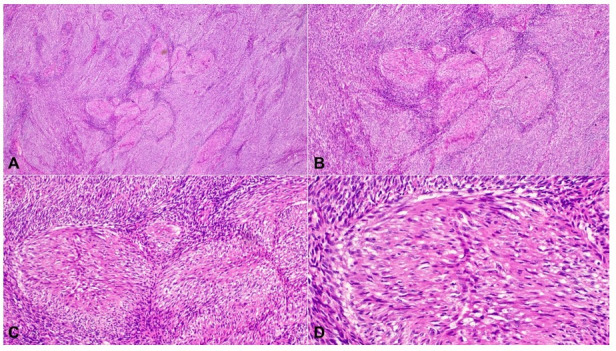
H&E sections showing areas of myoid differentiation in case 6. The cells contain brightly eosinophilic cytoplasm, well-defined cytoplasmic margins and vesicular nuclei. (**A**: 20x; **B**: 40x; **C**: 100x; **D**: 200x).

**Figure 2:**
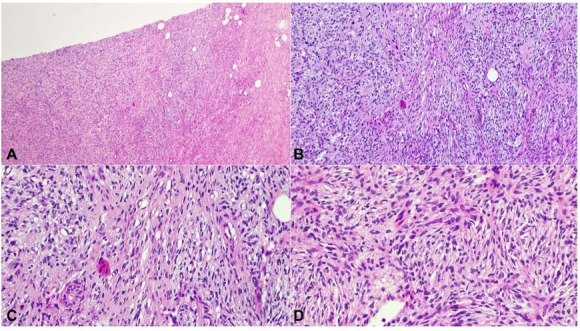
H&E sections showing areas of myoid differentiation in case 8. The myoid areas are diffuse and poorly demarcated. They are arranged in short fascicles. (**A**: 40x; **B**: 100x; **C**: 200x; **D**: 200x).

All cases were reviewed to confirm the diagnosis of DFSP and reassessed for myoid differentiation. The typical histology of DFSP is cytologically uniform, spindle cells with plump or elongated nuclei in storiform, whorled or cartwheel growth patterns.^1^ They typically infiltrate the subcutaneous fat in a honeycomb appearance. Cytological atypia is minimal and mitotic activity is low. While for fibrosarcomatous DFSP, the tumor often shows a nodular growth pattern and is composed of cellular spindle cell fascicles with a herringbone appearance, and increased cytological atypia and mitotic activity.^1^ Myoid differentiation is defined as tumor cells with brightly eosinophilic cytoplasm, well-defined cytoplasmic margins and vesicular nuclei. In our series of ten cases, case 6 (the only case of fibrosarcomatous DFSP) and case 8 were found to have myoid differentiation. In case 6, around 10% of the tumor area showed myoid differentiation (Figure 1). The myoid cells were arranged in confluent nodules and were in close proximity with vessels. In case 8, around 5% showed myoid differentiation (Figure 2). The myoid areas were diffuse and poorly demarcated. They formed short fascicles and were not associated with vessels. The details of the ten cases are listed in Table 1.

**Table 1:** Details of the cases in this study

**Case**	**Age**	**Sex**	**Location**	**Fibrosarcomatous variant**	**Myoid differentiation**	**FISH for COL1A1-PDGFB fusion**
1	16	F	AW	no	no	not done
2	25	F	breast	no	no	yes
3	25	M	thigh	no	no	yes
4	28	F	thigh	no	no	not done
5	41	F	AW	no	no	not done
6	47	M	back	yes	yes	not done
7	48	M	AW	no	no	not done
8	58	M	back	no	yes	not done
9	67	M	back	no	no	yes
10	75	M	back	no	no	yes

AW= abdominal wall, F= female, M= male.

**Figure 3:**
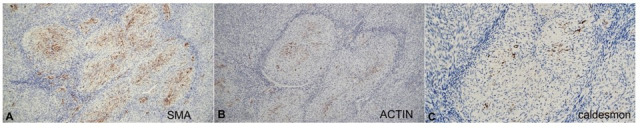
Immunostains: **A** – smooth muscle actin in areas of myoid differentiation in case 6, showing positive staining (100x); **B** – muscle specific actin in areas of myoid differentiation in case 6, showing patchy staining (100x); **C** – caldesmon in areas of myoid differentiation in case 6, showing a few positive cells (200x).

**Figure 4:**
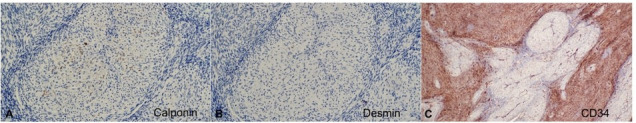
Immunostains: **A** – calponin in areas of myoid differentiation in case 6, showing a few positive cells (200x); **B** – negativity for desmin in areas of myoid differentiation in case 6 (100x); **C** – CD34 in case 6. The myoid areas are negative, in contrast to the adjacent classical areas showing diffuse positivity (20x).

Immunostaining for smooth muscle actin (SMA) was performed on all ten cases. The eight cases without myoid differentiation were negative, while the two cases with myoid differentiation (case 6 and 8) showed positivity in the myoid areas. Further immunostaining was performed in these two cases, including CD34, muscle-specific actin, caldesmon, calponin, and desmin (Figures 3 and 4). The results are listed in Table 2.

**Table 2:** Staining results in myoid areas

**Case**	**SMA**	**Muscle specific actin**	**caldesmon**	**calponin**	**desmin**	**CD34**
**6**	+	+	weak	weak	-	-
**8**	+	-	-	-	-	weak

**Figure 5:**
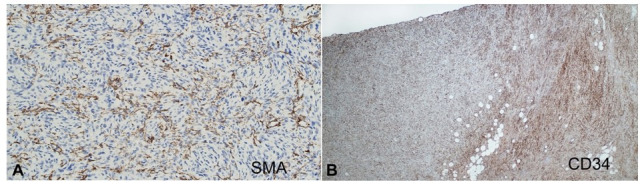
Immunostains **A** – smooth muscle actin in areas of myoid differentiation in case 8. The cells show patchy positivity (200x); **B** – CD34 in case 8 show weaker staining in myoid areas (left side) compare to the classical area (right side) (40x).

**Figure 6:**
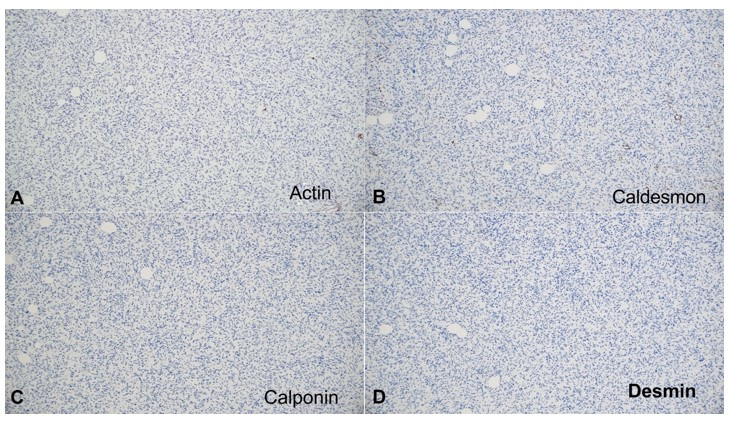
Immunostain: **A** – Areas of myoid differentiation in case 8 are negative for muscle specific actin(100x); **B** – Areas of myoid differentiation in case 8 are negative for caldesmon (100x); **C** – Areas of myoid differentiation in case 8 are negative for calponin (100x); **D** – Areas of myoid differentiation in case 8 are negative for desmin (100x).

For case 6 (fibrosarcomatous DFSP), the myoid areas were negative for CD34, while positive for muscle-specific actin and SMA. For case 8 (case without fibrosarcomatous differentiation), the myoid areas showed weaker staining for CD34 compared to the adjacent areas with classical morphology (Figure 5 and 6).

## DISCUSSION

The diagnosis of DFSP usually relies on the classical morphology and CD34 positivity. The differential diagnoses for classical DFSP include dermatofibroma (fibrous histiocytoma) and solitary fibrous tumor. Dermatofibromas are usually smaller lesions and more superficially located. Some may extend into the subcutis, but usually in a wedge-shaped pattern instead of a honeycomb pattern. The vast majority are negative for CD34. Solitary fibrous tumors are composed of ovoid to fusiform spindle cells arranged haphazardly or, in short, ill-defined fascicles, with characteristic staghorn (hemangiopericytoma-like) vessels. Since CD34 is sensitive but not specific for DFSP, the solitary fibrous tumor might be misdiagnosed as DFSP as both are positive for CD34, especially when those characteristic staghorn (hemangiopericytoma-like) vessels are not apparent in small biopsy samples. STAT6 is a solution as it is a highly sensitive and specific marker for solitary fibrous tumors.

Myoid differentiation is uncommon in DFSP, and more often found in fibrosarcomatous DFSP.^12^^,^^13^

The nature of the myoid cells is uncertain. Some suggested that they are likely to be related to hyperplasia of myofibroblasts in stroma rather than myofibroblastic differentiation of tumor cells based on its association with blood vessels.^14^ While some suggested that this might represent fibroblastic/myofibroblastic line of differentiation of tumor cells.^12^

In our study, two out of ten cases show focal myoid differentiation, including the only case of fibrosarcomatous DFSP. In the case of fibrosarcomatous DFSP, the myoid areas form confluent nodules, and are in close proximity with vessels. While in the case of usual DFSP, the myoid areas are more diffuse and mingled with the surrounding areas with classic morphology. This indicates that myoid differentiation may show different architecture, and the appreciation of cells with brightly eosinophilic cytoplasm, well-defined cytoplasmic margins and vesicular nuclei is the key to the correct diagnosis. Nevertheless, what these 2 cases share in common is that myoid differentiation Is a focal change (10% in of fibrosarcomatous DFSP and 5% in usual DFSP). The presence of classic DFSP morphology in other areas can aid the diagnosis. The myoid areas in DFSP can show positive staining (albeit patchy to focal) for smooth muscle markers, such as SMA, muscle-specific actin, caldesmon, and calponin. Staining for CD34 can be weak or even negative. These may create a diagnostic pitfall towards smooth muscle tumors or myofibroblastic lesions (e.g. leiomyoma, leiomyosarcoma, myofibroma), especially in small biopsy samples where areas of classic DFSP morphology may not be sampled. Especially for fibrosarcomatous DFSP with myoid differentiation, cutaneous leiomyosarcoma may also be considered as one of the differential diagnoses. Hence, recognition of myoid differentiation in DFSP is important. Nevertheless, the cells in leiomyoma and leiomyosarcoma are usually arranged in fascicles, with brightly eosinophilic cytoplasm. The typical honeycomb pattern of DFSP is not observed. They are usually diffusely and strongly positive for smooth muscle markers, and negative for CD34.

Although there is no specific immunostain to aid the diagnosis of DFSP, there is characteristic chromosomal translocation t(17;22)(q22;q13) in the large majority of DFSP. It is a fusion gene involving the collagen type 1 alpha 1 *(COL1A1*) gene on chromosome 17 and the platelet-derived growth factor β (*PDGFB*) gene on chromosome 22. The detection of *COL1A1-PDGFB* fusion by fluorescence in situ hybridization can be used to confirm the diagnosis, especially in histologically challenging cases.^15^^,^^16^

## CONCLUSION

Dermatofibrosarcoma protuberans (DFSP) is a relatively rare, locally aggressive and dermal based fibroblastic tumor. Fibrosarcomatous DFSP is an important variant as it acquires metastatic potential. Myoid differentiation in DPSP is rare, and more often found in fibrosarcomatous DFSP.^12^^,^^13^ The recognition of myoid differentiation is important as it may cause diagnostic difficulty with other tumors, especially in small biopsy samples. In histologically challenging cases, detection of *COL1A1-PDGFB* fusion by fluorescence in situ hybridization is helpful.
